# Cases Illustrating the Operation of the Eau Medicinale; from by Dr. Jones
*This gentleman, to whom the profession is indebted for the first, and we believe only detailed, account of the Eau Medicinale, has just returned from the Continent, and has communicated to Mr. Want some recently-acquired facts respecting this medicine, which will satisfy any remaining doubts that may exist relative to its identity with the Tincture of Colchicum. As we hope to present the public with this interesting document from the pen of Dr. Jones himself, as soon as his avocations will permit, we forbear to enlarge upon the subject.


**Published:** 1814-12

**Authors:** J. Want


*For the Medical and Physical Journal.*

### *Cases illustrating the Operation of the Eau Medicinale; from* by Dr. JONES.*

THE cases of Dr. Jones not being drawn up in subservience to any particular theory, afford a most unsuspected testimony of the manner in which the French remedy removes the paroxysm of Gout. I have thought it useful to extract the material facts related by him on this subject, principally with the view of confirming what I have before maintained from my own experience, that *purging* is not essential to the efficacy of that medicine.

The Earl of Essex took half a bottle, Thursday morning, fasting. By four in the afternoon the pain was considerably abated. He took the remaining part of the bottle going to bed,

* This gentleman, to whom the profession is indebted for the first, and we believe only detailed, account of the Eau Medicinale, has just returned from the Continent, and has communicated to Mr. Want some recently-acquired facts respecting this medicine, which will satisfy any remaining doubts that may exist relative to its identity with the Tincture of Colchicum. As we hope to present the public with this interesting document from the pen of Dr. Jones himself, as soon as his avocations will permit, we forbear to enlarge upon the subject.

bed, passed a comfortable night, and slept well; on Friday he awoke *without* pain. The medicine produced many evacuations that day, but it should be observed they had not taken place until the pain was entirely removed.

The Earl of Carlisle took half a bottle at bed-time; in a few hours felt relief, and in the morning was very free from pain. The medicine operated only as a sudorific, excepting only a slight nausea which it occasioned.

John Sands took a whole bottle; in a few hours was relieved, and slept comfortably; he awoke in the morning without pain. The operation of the medicine was powerful, by stool only. The time of its commencement is not specified.

Mr. Crow had taken forty drops of laudanum, without the slightest alleviation of his pain. The following night, going to bed, took a whole bottle; in a few hours considerably relieved; on awakening was free from pain. *Ten* hours after taking the medicine he had great nausea, followed by vomiting and copious bilious evacuations. As the pain was *quite* gone by on awakening in the morning, the operation on the bowels could not have taken place until after his amendment.

The Baron de Roll took a full dose of the eau medicinale at bed-time. The first part of the night was passed in great torture, but in a few hours he felt relief, and towards morning fell asleep: when he awoke, he was almost free from pain. The operation of the medicine was very trifling, and without the least disturbance. In two subsequent returns of the disease, it was removed in the same easy and expeditious manner, before the very moderate operation on the bowels began.

The Hon. Stewart Wortley took a *full* dose of the medicine at *night*. The pain was *soon* alleviated, and he rested comfortably. In the morning he was most agreeably surprised to find, that, although for some days he could not put his feet to the ground, he could now walk down stairs with ease. After all this relief, effected *before* the morning, nay very early during the night, it was not until the next day that he had considerable nausea, vomiting, and copious evacuations during all that day.

Mrs. ——— took half a bottle at bed-time, while suffering more severely than ever. Its effects were like those of enchantment. In three hours the pain had almost subsided, and she slept soundly. Slight nausea and two trifling evacuations were experienced, certainly not *before* the enchanting dismission of the pain.

Mr. Wood took a *whole* bottle at night. He slept soundly, and in the *morning* found the pain and swelling greatly abated.

Not

Not until he got out of bed, he felt a little sickness, which soon went off, after some moderate evacuations.

Viscount Morpeth took half a bottle at night, and he soon experienced relief. It produced a very copious perspiration, but *neither sickness nor evacuation.*

In Mr. Craufurd, the most violent attack, and which advanced to the greatest length, happened in January last. It came on suddenly in the night, in the most alarming and tremendous way. Agonizing pain seized the right side of the neck, the shoulder, and both hands and wrists. Before the hour of dinner the next day, he was unable to feed himself, or lift his arms off the cushions on which they were placed. At night the gouty swelling and inflammation were already apparent on the right wrist and elbow. Indications of the disease also began to appear in the knees and feet. Every thing, in short, portended a long and terrible fit, if suffered to run through its usual course. At bed-time he therefore took a full dose of the eau medicinale. In the morning the pain was greatly alleviated, and he could bear some motion of the affected limbs. By dinner-time he was able to make use of his hands, and on the third day the symptoms had totally disappeared. The remedy had no other perceptible effect on the stomach and bowels, than to excite a considerable degree of nausea, during the whole of the day after it was taken.

Mr. Harrison took a whole bottle. On *Monday morning*, he took half a bottle of the eau medicinale; in the evening, already felt relief, and took the remaining half. He enjoyed a good night's rest, and awoke on the *Tuesday morning* without pain. The medicine *now* began to operate in a *very gentle* way on the bowels.

Major Rennell. The medicine had no effect whatever on his stomach, and the slightest possible on the bowels. He felt an unusual degree of lassitude on the evening of the days on which he took the medicine, which might be owing to long fasting. It may, perhaps, be worth remarking, that on one of the days, when the inflammation in the hand was nearly gone, the eating of a moderate dinner (without wine) brought back the swelling and inflammation for some hours. It may be added, that on the first night he had the sweetest sleep that he had enjoyed for a length of time.

Sir Joseph Banks took half a bottle. The effect of it during the night was a gradual alleviation of pain, the gout first quitting the hand entirely, then the left shoulder, then the elbow, and so on. The next day the pulse was 62, and natural, the gout giving way in every joint. *Forty-eight* hours after the first dose, the remaining part of the bottle bottle was administered, and in the *course* of the next twelve hours, the medicine began to operate, and procured five evacuations. Until this time the medicine had no perceptible effect. In a fortnight afterwards, Sir Joseph was seized with a severe gouty lumbago. He had again recourse to half a bottle of the eau medicinale at bed-time, and before morning the pain and every other symptom were entirely removed. No mention is made of evacuation from the bowels, and, as the *same* dose of the medicine administered before was insufficient to produce this effect, setting aside Sir Joseph's positive declaration to me, it may be reasonably concluded that no such action did take place. At the first administration of the medicine, half a bottle had in some measure subdued the disease, which is described as then giving way in every joint, *without* purging, nor did the latter take place until a second dose was given *forty-eight* hours after the former, and then it required *twelve* hours to produce five evacuations—no great proof of the strong purgative quality of the medicine, and least of all of the necessity of that action to secure its curative effects.

Viscount Dillon took a *whole* bottle at night. The first effect was a profuse perspiration; in twelve hours was quite free from pain. It operated four times during the day *very moderately*, and without nausea and uneasiness. *A whole bottle operated very* moderately during the *day*, the medicine having been taken *over night*, and the pain removed within twelve hours, of course by the morning.

J. WANT.

